# Smartphone App–Based Noncontact Ecological Momentary Assessment With Experienced and Naïve Older Participants: Feasibility Study

**DOI:** 10.2196/27677

**Published:** 2022-03-08

**Authors:** Louise Burke, Graham Naylor

**Affiliations:** 1 Hearing Sciences - Scottish Section School of Medicine University of Nottingham Glasgow United Kingdom

**Keywords:** ecological momentary assessment, EMA, smartphone, mobile phone, mHealth, app, noncontact EMA, remote EMA, hearing, hearing loss, feasibility

## Abstract

**Background:**

Smartphone app–based ecological momentary assessment (EMA) without face-to-face contact between researcher and participant (*app-based noncontact EMA*) potentially provides a valuable data collection tool when geographic, time, and situational factors (eg, COVID-19 restrictions) place constraints on in-person research. Nevertheless, little is known about the feasibility of this method, particularly in older and naïve EMA participants.

**Objective:**

This study aims to assess the feasibility of app-based noncontact EMA as a function of previous EMA experience, by recruiting and comparing a group of participants who had never participated in EMA before against a group of participants who had been part of an earlier in-person EMA study, and age, by recruiting middle-aged to older adults.

**Methods:**

Overall, 151 potential participants were invited via email; 46.4% (70/151) enrolled in the study by completing the baseline questionnaire set and were emailed instructions for the EMA phase. Of these participants, 67% (47/70) downloaded an EMA app and ran the survey sequence for 1 week. In total, 5 daytime surveys and 1 evening survey, each day, assessed participants’ listening environment, social activity, and conversational engagement. A semistructured *exit* telephone interview probed the acceptability of the method. As markers of feasibility, we assessed the enrollment rate, study completion rate, reason for noncompletion, EMA survey response rate, and likelihood of reporting an issue with survey alerts and requested assistance from researchers, family, or friends.

**Results:**

Enrollment rates among invitees (63.3% vs 38.2%; *P*=.004) and completion rates among enrollees (83.9% vs 53.8%; *P*<.001) were higher in the experienced than in the naïve EMA group. On average, experienced participants responded to 64.1% (SD 30.2%) of the daytime EMA surveys, and naïve participants responded to 54.3% (SD 29.5%) of the daytime EMA surveys (*P*=.27). Among participants who retrospectively reported issues with survey alerts, only 19% (3/16) requested researcher assistance during data collection. Older participants were more likely to report not being alerted to EMA surveys (*P*=.008), but age was unrelated to all other markers of feasibility. Post hoc analyses of the effect of the phone operating system on markers of feasibility revealed that response rates were higher among iOS users (mean 74.8%, SD 20.25%) than among Android users (mean 48.5%, SD 31.35%; *P*=.002).

**Conclusions:**

Smartphone app–based noncontact EMA appears to be feasible, although participants with previous EMA experience, younger participants, and iOS users performed better on certain markers of feasibility. Measures to increase feasibility may include extensive testing of the app with different phone types, encouraging participants to seek timely assistance for any issues experienced, and recruiting participants who have some previous EMA experience where possible. The limitations of this study include participants’ varying levels of existing relationship with the researcher and the implications of collecting data during the COVID-19 social restrictions.

## Introduction

### Background

Ecological momentary assessment (EMA) refers to a range of methods used for measuring daily life feelings, events, experiences, and behaviors in real-time, real-world settings [1]. These methods include pen-and-paper surveys and diary methods [2,3] and surveys delivered via palmtop computers [4,5], mobile phone SMS text messaging [6,7], and, most recently, smartphone apps [8-10]. In the past decade, smartphone app–based EMA has surged in popularity, coinciding with an exponential growth in smartphone ownership; in the United Kingdom, 87% of the population owned a smartphone in 2020 compared with only 27% in 2010 [11,12]. This presents an opportunity for participants to partake in app-based noncontact EMA, where they can download an app and run EMA schedules from their own smartphone, without the need for in-person interaction with researchers at study invitation, recruitment, initiation, or data collection.

Among its advantages, app-based noncontact EMA allows for the recruitment of large samples, or those drawn from specific or hard-to-reach populations, more easily than in-person EMA. It is convenient and can reduce or remove time and geographic barriers to participation. The participant burden associated with borrowing, becoming familiar with, and carrying around a supplied research smartphone is eliminated. For researchers, equipment costs are reduced, and allowing participants to use their own personal smartphone means that less training is required, thus saving time. When training is provided, it is more feasible to use a blanket approach (eg, distribution of generic written instructions only) with noncontact EMA than in-person EMA. Finally, noncontact EMA presents an alternative when situational factors, such as the COVID-19 restrictions, prevent in-person research.

However, there are also limitations associated with this method, not least that participants must own a smartphone. Furthermore, EMA schedules can be complex and demanding, and therefore require a certain degree of participant investment and a solid understanding among participants of what they are being asked to do, along with technological proficiency with the smartphone and app. This may be difficult to achieve without an in-person initiation. Finally, in allowing participants to run EMA on their own smartphones, researchers have less control over the study, and the wide variety of smartphone devices on the market makes it difficult to provide technical support to participants when required. These factors may have negative implications for the quality and quantity of the EMA data collected.

One may reasonably expect that previous experience of successful participation in EMA studies (in whatever form) would increase the likelihood of being able or willing to complete a subsequent app-based noncontact EMA study, although to the authors’ knowledge, this has never been explored. In contrast, participants who are new to the EMA method may be disadvantaged by the lack of in-person initiation and subsequently struggle with the noncontact EMA protocol. Regarding the potential effects of age, the high technological demand of EMA may prove challenging for older adults who tend to have lower rates of smartphone ownership, use [13], and competency [14]. Notably, Duncan et al [15] found that, among a sample of sexual minority men, older participants were less willing to download the EMA app to their smartphone than younger individuals. Hence, the limitations of app-based noncontact EMA may lower its feasibility for older participants and introduce sampling bias. As app-based noncontact EMA has become necessary under the COVID-19 restrictions, it is essential to understand the feasibility of this method.

### Existing Evidence of Feasibility

To explore the prevalence of published app-based noncontact EMA research and uncover any evidence relating to the abovementioned effects of age and EMA experience, a literature search of the Scopus database was conducted. The search was conducted in April 2020, using the terms *ecological AND momentary AND assessment AND smartphone.* A total of 404 articles were found, all published from 2011 onward. After removing duplicates and inaccessible or irrelevant articles, 73.3% (296/404) of studies remained. Of the 296 studies, 220 (74.3%) used in-person recruitment or initiation techniques, and the studies described by 47 articles were indeterminable as in-person or noncontact EMA. In total, 29 articles reported noncontact procedures, and all were published since 2015. Of these 29 articles, 3 (10%) were protocol papers and 2 (7%) used SMS text message–based sampling measurements. Therefore, 83% (24/29) of publications described observational app-based noncontact EMA studies, demonstrating that this method has been enjoying minimal (in contrast to in-person EMA) but increasing application in recent years.

A meta-analytic approach was used to determine the characteristics of the 24 studies. The median sample size across the studies was 135 participants (range 17-6675). Among the studies which reported mean age and dichotomous male–female gender distribution of the sample, participants tended to be younger (mean 30.9, SD 8.0 years, based on 21 studies) and mostly women (mean 68.7% of the sample, based on 19 studies). The topics of study were smoking [16,17], alcohol consumption [18-22], mental health [23-29], dietary behavior [30-35], drug use [36,37], physical activity [38], and tinnitus management [39].

All studies used noncontact methods of recruitment, initiation, and EMA data collection, although the specific procedures varied across studies. Advertisement was conducted on the web via circulation of study details on social media [28,39] and within Reddit and other web-based communities [27,29], and offline via mass media [26,28], flyers [30], and networking or word of mouth [18,22,30]. When an existing database of university students was available, email invitations were used to advertise studies and recruit samples [21,35,38]. Recruitment and initiation of participants commonly involved directing participants to a webpage where they could read the study description, provide informed consent, complete baseline measures, and access instructions for downloading and using the EMA app [18,33]. In some studies, written instructions for the EMA phase were supplemented by a telephone call [31] or a prerecorded video demonstration [35], indicating that noncontact initiation is more intensive in some studies than others.

Although the specifics of EMA data collection varied depending on the purpose of the study, all 24 studies required participants to run the app on their own smartphone; some specified that this must be running iOS [19] or Android [24], whereas others allowed both [22]. In most studies, the feasibility of this aspect of the design was unaddressed, although McQuoid et al [37] reported that iOS and Android smartphones collected sensor data at different rates in their EMA study; Schlee et al [39] noted low controllability owing to participants using their own smartphones as a limitation of their study, and Wouters et al [35] concluded that the main cause of participant dropout in their study was related to a technical issue caused by an Android update during data collection. Notably, participant dropout rates were not systematically reported; in 71% (17/24) of the studies, it is implied, but not explicitly stated, that no dropout occurred, whereas some reported dropout rates ranging from 14% to 37% [22,23,32-35,38]. Finally, across the 6 studies that did not exclude participants based on low response rate and reported response rate as a percentage of delivered signal-contingent EMA surveys [18-21,32,39], the mean response rate was 65%.

None of the aforementioned 24 studies directly assessed the feasibility of app-based noncontact EMA in comparison with in-person EMA. To the authors’ knowledge, the only study to date to have done so is that of Carr et al [40], which was not published when we conducted the literature search. In their sample of men who have sex with men (N=100; mean age 27 years), participants were enrolled in the study and were initiated either in-person or via a live videoconferencing session. The measured outcomes included response rate, behavioral reactivity, and consistency and reliability of the EMA responses. The in-person and noncontact groups returned similar response rates, similar levels of behavioral reactivity, and equally consistent and reliable EMA responses. This suggests that app-based noncontact EMA is feasible and equivalent to EMA initiated in-person, at least with younger, male participants who are initiated via videoconferencing. The use of videoconferencing by Carr et al [40] to initiate noncontact participants means that their findings may not be generalizable to studies that use written instructions [15-17]. Indeed, videoconferencing might arguably be classified as *in-person* for the purposes of instruction and initiation.

### Study Objectives

From the foregoing, it is apparent that much remains unknown regarding the feasibility of app-based noncontact EMA. Specifically, evidence is lacking regarding the method’s feasibility with older adults and with experienced versus naïve EMA participants. Therefore, the research questions are as follows:

1. How does the feasibility of app-based noncontact EMA compare, for participants who have never participated in EMA before versus participants who have previous in-person EMA experience?
2. Does older age have an adverse effect on the feasibility of app-based noncontact EMA?

Given the paucity of previous evidence relating to these questions, this study adopted an exploratory approach rather than testing specific hypotheses.

## Methods

### Participants and Recruitment

In total, 151 members of our pre-existing participant pool were invited. Recruitment occurred in 2 stages. First, 32.5% (49/151) of people who had participated in an earlier in-person EMA study of listening and daily life fatigue conducted by Burke and Naylor [41] (referred to hereafter as the EMA Fatigue study) were invited. In the study, which ran 12 to 18 months before this study, participants were selected randomly from our participant pool, invited by postal letter, and attended three in-person sessions: an initiation session at baseline, a check-in session midway through the study, and a debriefing and interview session at the end. They used smartphones supplied by the researchers with the EMA app preinstalled (the same app as used in this study) to respond to 6 EMA surveys per day for 2 weeks. In all, 50.3% (76/151) of participants initiated the study, and 44.4% (67/151) completed the study. The participants who initiated but subsequently dropped out of that study were not invited to this study to reduce variation with respect to the level of EMA experience acquired. Of the 67 participants who completed the EMA Fatigue study, 18 (27%) were no longer contactable. Thus, 49 individuals with previous EMA experience of 2 weeks, including in-person initiation, were invited to participate in this study. Of these, 63% (31/49) accepted the invitation and formed the *experienced* EMA group.

Thereafter, an additional sample of 102 participants was invited. They had not participated in the EMA Fatigue study, although 19.6% (20/102) were selected randomly and invited to participate in that study—an overlap that resulted from sampling from the same participant pool for both studies. Recruitment of this *naïve* EMA group was conducted on a rolling basis to recruit a group that was similar in size, age, and gender distribution to the experienced EMA group. Initially, 26 invitations were sent, followed by 30 more, followed by 46 more, at which point the naïve group matched the experienced group to an acceptable extent. Of the 39 who accepted the invitation, all but one reported that they had not previously participated in smartphone-based research; one was unsure.

As the aim of recruitment was to recruit as many *experienced* participants as possible from a finite group of past participants, and subsequently to recruit a similarly sized naïve group, this drove our recruitment approach rather than power analyses, which would have been redundant. Notably, all members of our participant pool were recruited from the National Health Service Audiology in Glasgow, although not all experience hearing loss; issues of tinnitus, hyperacusis, and balance, for example, are also present within our participant pool. The only inclusion criterion was that participants had to own either an Android or iOS smartphone. Invitees’ smartphone ownership status was unknown before recruitment.

### Ethical Approval

Ethical approval was received from the West of Scotland Research Ethics Committee (18/WS/0007) and the National Health Service Research and Development (GN18EN094). This study was not preregistered.

### Procedure

Data were collected from June to August 2020. Email invitations included a participant information sheet and a link to the web-based consent form and baseline questionnaire set. The participants who provided consent and completed the questionnaires were sent a follow-up email containing *beginning the study* and *finishing the study* instruction guides. These participants were asked to download the EMA app to their smartphone and run our EMA survey sequence, which was launched when participants completed a *start-up* survey. At this point, they received an email confirming their enrollment in the study, start and end dates of the EMA phase, and the date and time to expect a telephone *exit* interview. Participants who provided consent and completed the baseline questionnaire set, but did not run the EMA sequence, were emailed a link to a follow-up web-based survey that asked them why they had failed to complete the study.

The EMA sequence consisted of 7 full days of smartphone surveys. Each day, 5 *daytime* surveys were received randomly between 9 AM and 8 PM, with at least an hour between the surveys. Participants had up to 30 minutes to respond to the daytime surveys before they expired. One *evening* survey was received at 9 PM each night, and participants had up to 3 hours to respond. On the final day of the EMA data collection, participants received a *final* survey at 8:30 PM, instead of an evening survey. This informed participants that they had finished the EMA sequence and advised them to consult the *finishing the study* instruction guide, which instructed them to check their data upload status within the app, upload data if necessary, and uninstall the app. The final survey did not expire, so the response window was unlimited.

Telephone *exit* interviews were conducted with all participants who ran the EMA sequence. Participants were made aware of their invitation email that they would be compensated a fixed amount, a £20 (US $27) Amazon e-voucher, to complete the study. They were not paid on a per-survey basis; therefore, there was no monetary incentive to complete the EMA surveys. Participants who completed the baseline survey, 1-week EMA sequence, and telephone interviews received full compensation. It was decided after the data collection, to compensate participants who only completed the baseline survey with a £10 (US $13.5) Amazon e-voucher.

### Materials

The web-based consent forms and baseline questionnaires were created and administered using the JISC Online Surveys [42]. The LifeData EMA platform [43] and its corresponding smartphone app, RealLife Exp [44], were used to create and administer smartphone EMA surveys. All the participants used their own smartphones. A participant information sheet explaining the study in detail, a *beginning the study* instruction guide, explaining how to install and use the app and respond to EMA surveys, and a *finishing the study* instruction guide, containing information about data upload and app uninstallation, were supplied as PDF documents.

### Measures

#### Demographic Information

Invitees’ age (years), gender (*male, female, or prefer not to say*), degree of hearing loss (represented as better ear average pure-tone threshold across four frequencies: 500 Hz, 1 kHz, 2 kHz, and 4 kHz), and the number of previous in-person appointments attended at our facility were retrieved from the participant database.

#### Baseline Questionnaires

In all, 3 questionnaires were administered at baseline and are described further. As the study was conducted during the COVID-19 restrictions, some of the questionnaires were modified to better reflect those circumstances. These questionnaires were not directly related to the research questions being examined in this paper; in this context, they merely serve as a procedural step which is often found in EMA studies, namely acquisition of descriptive variables at baseline. As this is a step at which participants may drop out, its presence is relevant for the current purposes. Its content is not relevant, beyond assessing age associations and whether the naïve and experienced groups were equivalent on the variables collected.

An adapted Hearing Handicap Inventory for Adults/the Elderly [45,46] measured the perceived social and emotional consequences of hearing loss. This comprises 26 items and takes approximately 7 minutes to complete. The social activity log [47] was adapted to measure social activity during the past week and month using 15 items and took approximately 5 minutes to complete. Finally, the 10-item Technology Readiness Index (version 2.0 [TRI 2.0]) [48] measured attitudes toward and adoption of technology in the home. A mean value between 1 and 5 was computed for each participant, with higher values reflecting higher levels of techno-readiness. This questionnaire takes approximately 3 minutes to complete. The TRI 2.0 has been found to be both a valid and reliable measurement tool [48].

#### EMA Surveys

##### Start-up Survey

A 1-time start-up survey elicited participants’ employment status (collapsed into *full- or part-time employed* or *retired/not working/familial caregiver*) and whether they had participated in smartphone-based research previously (*yes*, *no*, or *unsure*).

##### Daytime Survey

The daytime survey (occurring 5 times per day) elicited self-reports of the type of location, presence or absence of other people, conversational situation, level of background noise, and length of time spent in the situation.

##### Evening Survey

The evening survey consisted of 6 questions regarding participation in and avoidance of social activity, conversational engagement, and hearing difficulty during that day.

All EMA surveys logged the make, model, and operating system of the phone used to respond.

##### Follow-up Survey

Participants who completed the baseline questionnaires, but failed to proceed further, were emailed a short JISC survey exploring the reasons for noncompletion of the study. Prefixed by “I did not download the app because,” response options were (1) “I did not receive any instructions to do this,” (2) “The instructions looked too complicated,” (3) “I tried to download the app but found it too difficult/did not know how,” (4) “I did not want to,” (5) “I did not have time/I was too busy,” (6) “I did not realize this was part of the study,” (7) “I do not own a smartphone,” (8) “My smartphone would not download the app,” (9) “I have not got around to it yet,” and (10) “Other”. Participants were permitted to select only 1 response option.

##### Exit Interview

The exit interview explored the acceptability of the set-up process (eg, ease of installing the app and completing the start-up survey), study participation (eg, satisfaction with auditory survey notifications and size of question text), procedure for ending the study (eg, data upload status and uninstallation of the app), and general topics (eg, use and usability of instruction guides and reactions of family and friends). In addition, participants with previous EMA experience were asked if they preferred using their own smartphone, as in this study, or a supplied smartphone, as in the EMA Fatigue study.

### Data Analysis

#### Predictor Variables

There were two primary predictors in this study: previous EMA status and age. Participants were classified as experienced EMA participants if they had taken part in the EMA Fatigue study and as naïve EMA participants if they had not. These classifications were corroborated by participant responses to the question which asked if they had previously taken part in any smartphone-based research. Age (years) was treated as a continuous predictor. Interview responses, specifically the high proportion of Android users reporting issues with survey alerts, prompted the post hoc addition of one secondary predictor: phone operating system (coded as *Android* or *iOS*).

#### Outcome Variables: Markers of Feasibility

The outcome variables were the markers of the feasibility of the app-based noncontact EMA. These were (1) enrollment rate, represented as the percentage of invitees who enrolled into the study by completing the baseline questionnaire set; (2) completion rate, represented as the percentage of enrollees who ran the EMA sequence for 1 week; (3) reason for noncompletion of the study among enrollees, coded dichotomously as *technical reason* or *personal reason*; and (4) EMA survey response rate, measured as the percentage of delivered signal-contingent (ie, daytime and evening) EMA surveys which were responded to. Interview responses prompted the creation of 1 post hoc outcome variable, that being (5) reported issue with survey alerts (coded as *yes* or *no*). Finally, (6) requested assistance from either the research team or a family member or friend, each coded dichotomously as *yes* or *no*, comprised another marker of feasibility. A final outcome measure that applied only to the experienced EMA group was preference for using personal phone or a supplied phone, coded as *preferred own phone*, *preferred supplied phone*, or *no preference*.

#### Statistical Techniques

First, the associations between the experience group and baseline factors were examined using independent-samples 2-tailed *t* tests and chi-square tests, and the associations between age and baseline factors were assessed using Pearson correlations and binary logistic regression. For all markers of feasibility, summary statistics (means and SDs for continuous variables and frequencies and percentages for categorical variables) were computed for the experienced and naïve groups, and comparisons were made using independent-samples *t* tests and chi-square tests. The associations between age and all markers of feasibility were examined using Pearson correlations and logistic regression analyses. The phone operating system was assessed as a predictor of marker 4 (response rate), and markers 6a and 6b (requested assistance from the research team and from family or friends, respectively), using independent-samples *t* tests and chi-square tests. Finally, responses regarding smartphone preferences were counted for the EMA group.

Across all analyses, chi-square tests were only conducted when the minimum cell count in each group was 5. The analyses were conducted using the SPSS Statistics (version 25; IBM Corp). The α level was set to .05, for all analyses.

## Results

### Sequence Completion and Markers of Feasibility

**Figure 1:**
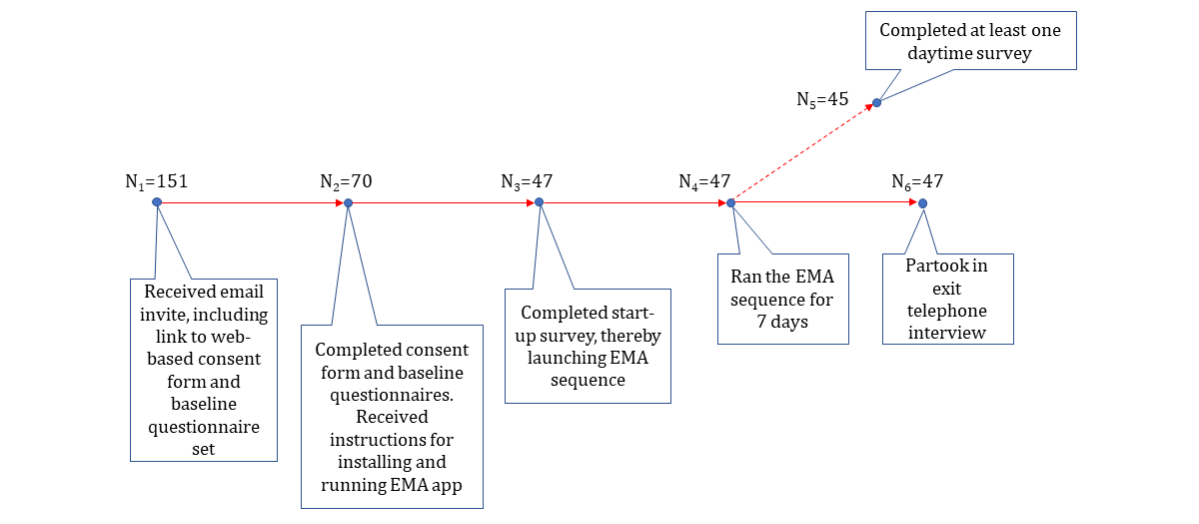
Participation at each stage of the study. EMA: ecological momentary assessment.

Figure 1 illustrates the flow of participants and the number of participants who reached various stages of the study.

### Experience Level

#### Group Characteristics and Differences at Baseline

The baseline characteristics and comparisons between the experienced and naïve EMA groups are presented in Table 1. The only baseline variable on which the groups differed was the number of previous laboratory appointments attended, with experienced EMA participants having attended more on average.

**Table 1:** Baseline characteristics and differences by experience group.

Characteristics	Experienced EMA^a^ group (n=26)^b^	Naïve EMA group (n=21)^b^	Group comparisons		
			Chi-square (*df*)	*t* test (*df*)	*P* value
Age (years), mean (SD), range	66.2 (8.6), 45-78	65.1 (8.6), 46-75	N/A^c^	−0.45 (45)	.65
Gender (male), n (%)	12 (46)	8 (38)	0.31 (1)	N/A	.58
Employed, n (%)	6 (23)	7 (33)	0.61(1)	N/A	.44
Degree of hearing loss in dB HL^d^, mean (SD)	22.7 (14.2)	22.1 (13.2)	N/A	−0.14 (45)	.89
Hearing aids users, n (%)	9 (35)	9 (43)	0.33 (1)	N/A	.56
Previous laboratory appointments attended, mean (SD)	5.2 (2.0)	3.3 (2.2)	N/A	−3.04 (45)	.004
HHIA/E^e^ score (hearing handicap), mean (SD)	32.3 (29.7)	25.0 (17.6)	N/A	−1.04 (39.88)	.30
SAL^f^ score (social activity), mean (SD)	1.9 (1.0)	2.0 (0.9)	N/A	0.34 (44)	.74
TRI^g^ 2.0 score (techno-readiness), mean (SD)	3.3 (0.7)	3.2 (0.7)	N/A	−0.14 (44)	.89
Android users, n (%)	15 (58)	12 (57)	0 (1)	N/A	.97

^a^EMA: ecological momentary assessment.

^b^Mean (SD) and *t* test values are presented for continuous variables and n (%) and chi-square values are presented for categorical variables.

^c^N/A: not applicable.

^d^dB HL: decibels in hearing level.

^e^HHIA/E: Hearing Handicap for Adults/the Elderly.

^f^SAL: social activity log.

^g^TRI: Technology Readiness Index.

#### Effect of Previous EMA Experience on Markers of Feasibility

As shown in Table 2, the enrollment rate (marker 1) was almost twice as high in the experienced EMA group compared with the naïve EMA group (*χ*^2^_1_=8.3; *P*=.004). Similarly, the completion rate (marker 2) was significantly higher among experienced enrollees compared with their naïve counterparts (*χ*^2^_1_=7.1; *P*=.008). In the experienced group, 60% (3/5) of the participants who enrolled in the study but did not complete it, provided a reason why (marker 3, the only marker not displayed in Table 2); 67% (2/3) cited technical difficulties and 33% (1/3) reported a personal reason. In the naïve EMA group, 44% (8/18) provided a reason why; 25% (2/8) cited technical difficulties and 75% (6/8) cited personal reasons. There were too few responses in each group to analyze group-wise differences in this outcome variable.

**Table 2:** Markers of feasibility in the overall sample, experienced ecological momentary assessment (EMA) group, and naïve EMA group.

Characteristics	Overall^a^	Experienced^b^	Naïve^c^
N_1_ (participants receiving study invitation)	151	49	102
N_2_ (participants initiating study)	70	31	39
Marker 1: enrollment rate among invitees (%; N_2_/N_1_)	46.4	63	38.2
N_3_ (participants launching EMA sequence)	47	26	21
N_4_ (participants running the 7-day EMA sequence)	47	26	21
Marker 2: completion rate among enrollees (%; N_4_/N_2_)	67.1	84	53.8
N_5_ (participants completing at least one daytime EMA survey)	45	25	20
N_6_ (partook in exit interview)	47	26	21
Marker 4: signal-contingent survey response rate (%), mean (SD)	61.4 (30.1)	65.4 (30.7)	56.3 (29.3)
Marker 5: reported survey alert issue (n, yes)	16	7	9
Marker 6a: requested assistance from researcher (n, yes)	3	2	1
Marker 6b: requested assistance from family or friends (n, yes)	6	2	4

^a^Overall sample.

^b^Experienced EMA group.

^c^Naïve EMA group.

The response rate on signal-contingent (ie, daytime and evening) EMA surveys (marker 4) ranged from 0% to 100% in the experienced EMA group and from 0% to 90.9% in the naïve EMA group. The response rate (as shown in Table 2) was not significantly different among the groups (*t*_45_=−1.04; *P*=.31). One participant in each group did not respond to any signal-contingent survey; both reported that they had not been alerted to the incoming surveys. In total, 16 participants reported not being alerted to surveys either some or all the time, as shown in Table 2. There was no effect of previous EMA experience on the likelihood of reporting this issue (*χ*^2^_1_=0.0; *P*=.93). Compared with those who did not report any issue with alerts (marker 5; mean 65.9%, SD 25.6%), those who had attained a lower average response rate (mean 47.7%, SD 34.7%*; t*_45_=2.04; *P*=.047).

In all, 3 participants contacted the researcher for help during the study (marker 6a), all in relation to survey alerts not working, and 6 participants reported asking for help from a family member or friend with some technical aspects of the study (marker 6b). There was too little variation in the responses to assess the effect of past experience on these outcomes.

### Age

#### Relationship of Age to Baseline Variables

Unsurprisingly, older age was related to a greater likelihood of being retired (Wald *χ*^2^_1_=10.0; *P*=.002), more severe hearing loss (*r*=0.31; *P*<.001), and a greater likelihood of being a hearing aid user (Wald *χ*^2^_1_=5.4; *P*=.02). Age was also related to gender (Wald *χ*^2^_1_=4.5; *P*=.04), such that older participants were more likely to be men. No association was found between age and the number of previous laboratory appointments attended (*r*=0.29; *P*=.05), hearing handicap (*r*=−0.10; *P*=.50), social activity (*r*=0.06; *P*=.67), techno-readiness (*r*=0.04; *P*=.79), or the likelihood of being an Android (vs iPhone) user (Wald *χ*^2^_1_=0.0; *P*=.90).

#### Effect of Age on Markers of Feasibility

Age was unrelated to both enrollment rate (marker 1; Wald *χ*^2^_1_=0.7; *P*=.39) and completion rate (marker 2; Wald *χ*^2^_1_=0; *P*=.99). The effect of age on the reason for noncompletion (marker 3) was not assessed, as too few responses were obtained. Age was unrelated to response rate (marker 4; *r*=0.19; *P*=.21), but older participants were more likely to report not being alerted to surveys (marker 5; Wald *χ*^2^_1_=7.0; *P*=.008). The effect of age on the likelihood of requesting assistance (markers 6a and 6b) was not examined because of the homogeneity of responses.

### Phone Operating System

The phone operating system was related to response rate (marker 4); in comparison with Android users, participants using iOS returned a higher response rate (mean 74.8%, SD 20.3% vs mean 48.5%, SD 31.4%; *t*_45_=−3.28; *P*=.002). Among iOS users, 25% (5/20) reported not being alerted to surveys (marker 5) compared with 41% (11/27) of Android users; however, this difference was not statistically significant (*χ*^2^_1_=1.3; *P*=.26). All 3 participants who sought assistance from the research team (marker 6a) were Android users. Of the 6 who reported asking a family member or friend for help (marker 6b), 4 (67%) were Android users and 2 (33%) were iOS users. The effect of the phone operating system on markers 6a and 6b was not assessed because of the lack of variability of responses on these outcomes.

Although both older age and using an Android operating system were related to reporting issues with survey alerts, the mean age of Android users in this study (mean 65.9, SD 7.9 years) was not significantly different from that of iOS users (mean 65.6, SD 9.4 years; *t*_45_=0.12; *P*=.91).

### Experience of Using Personal Versus Supplied Smartphone

The 26 experienced EMA participants who completed the study were asked if they preferred using their own smartphone, as in this study, or using a supplied smartphone, as in the EMA Fatigue study. Of these 26 participants, 22 (85%) preferred using their own phone, whereas 4 (15%) preferred using the supplied research phone and/or preferred the face-to-face method in general.

## Discussion

### Principal Findings

This study aimed to assess the feasibility of noncontact EMA. To the authors’ knowledge, this is only the second study to do so, after Carr et al [40], and is the first to examine the effects of previous EMA experience, age, and phone operating system on the feasibility of this method. Although the findings suggest that previous in-person EMA experience, younger age, and iOS use may be advantageous, the observed effects were specific to certain markers of feasibility and not systematic across all possible indicators. It is therefore suggested that app-based noncontact EMA is feasible to run with naïve and older EMA participants.

Perhaps the most compelling finding from this study is that naïve participants were less likely to enroll in and complete the study than their experienced counterparts. However, confounding effects could not be ruled out. Specifically, experienced EMA participants had previously attended more in-person laboratory appointments than naïve participants, which may indicate a stronger degree of existing relationship with the researcher or greater willingness to participate in research, among these participants. Future research would benefit from the measurement and tight control of these factors. The observed higher rate of attrition among naïve participants between the baseline survey and EMA phase is concerning. This may indicate technological difficulties in downloading the app or a lack of understanding of the study demands. A low response to the follow-up survey among dropouts means that little information has been gathered regarding the reasons for attrition; therefore, the feasibility of app-based noncontact EMA in this respect remains unclear. Notably, age was unrelated to both enrollment and completion rates, suggesting that the feasibility of this method is not sensitive to age (within the range studied here; 45-78 years).

Turning to the responses to individual survey alerts, it is notable that the response rate was unrelated to both previous EMA experience and age. At 61.4%, the response rate in this study corresponds closely to the 65% mean of response rates across other noncontact EMA studies [18-21,32,39]. We may have expected a higher response rate, given that this study was conducted at a time when many people were confined to their homes and unable to work, travel, or attend social gatherings owing to the COVID-19 pandemic. Hence, some of the main reasons for nonresponse to EMA surveys, including being in a noisy environment and not hearing the alert, or being in a social situation where it would be inappropriate to respond [41,49], were less likely to occur. However, our participants were not paid per EMA survey completion, as in some other EMA studies [27,29], and it is also possible that participants’ motivation to respond about their social interactions was reduced when social activity was restricted. Overall, this study yielded acceptable response rates.

Finally, a sizable number of participants reported that they were not alerted to some or all EMA survey alerts, and their response rates were unsurprisingly lower than those returned by unaffected participants. Whether participants truly experienced a technical issue, or were just unaware of alerts, cannot be determined by the data. However, older participants were disproportionately affected by this issue. These findings undermine the feasibility of app-based noncontact EMA and suggest that supplying participants with a preprogramed smartphone (which would typically involve an in-person initiation session) yields better results in terms of data quantity. However, participants in this study who had experience using both their own smartphone and a supplied research smartphone for EMA overwhelmingly preferred using their own. Future app-based EMA research (both in-person and noncontact) should consider giving participants the option to use either their own smartphone or a supplied smartphone where possible [50], and the chosen app should be tested extensively with different phone makes, models, and operating systems.

### Limitations

In addition to caveats mentioned above with respect to specific results, there are several more wide-ranging factors which may limit the reliability and generalizability of this study’s results.

First, all participants had working email accounts, possibly indicating some level of technological competence. In support of this, the mean TRI 2.0 score obtained by this sample was slightly higher than that reported by the Parasuraman and Colby [48] representative sample of US-based adults, reported in the paper in which they introduced the TRI 2.0 (3.33 units vs 3.0 units on a 1-5 Likert scale). Moreover, all participants were recruited from our participant pool, meaning that they had attended at least one in-person appointment at our facility and therefore had some degree of existing relationship with the researchers. This is also a strength of the study, as it indicates some level of homogeneity of participant background, all having come from Glasgow National Health Service Audiology. However, these factors limit the generalizability of our findings. In addition, in the experienced group, the decision not to invite participants who had dropped out of the EMA Fatigue study to recruit a genuinely *experienced* EMA group in this study may have led to the recruitment of an unrepresentatively motivated or conscientious group.

Limited variation and lack of data pertaining to some of the markers of feasibility in this study suggest that they are not useful outcomes. Furthermore, despite attempts to explore the reasons for noncompletion of the study by sending a follow-up survey to the dropouts, only few participants responded. Even less is known about why individuals declined to participate in this study in the first place. Crucially, the smartphone ownership status of this group is not known, a key factor in terms of feasibility.

Finally, our method was noncontact insofar as participants did not attend in-person laboratory sessions; however, they did receive detailed instructions to guide them through the study and were contacted several times by email during recruitment, initiation, and enrollment, as detailed in the *Procedure* section. Consequently, the findings are most relevant to physical noncontact, but nonetheless interactive, EMA research.

### Conclusions

This study assessed the feasibility of app-based noncontact EMA as a function of past EMA experience and age. Experienced EMA participants were more likely to enroll in and complete the study than naïve participants, whereas age was unrelated to both enrollment and completion rates. The response rate was acceptable and unrelated to both experience and age, but Android users returned markedly lower response rates than iOS users. Although a sizable number of (mostly older) participants reported in exit interviews that they were not always alerted to surveys, very few informed or sought assistance from the researchers during data collection. In summary, app-based noncontact EMA is feasible, although consideration should be given to how to increase enrollment and completion rates, especially when recruiting participants who are new to EMA.

## Abbreviations

EMA: ecological momentary assessment
TRI 2.0: Technology Readiness Index (version 2.0)
